# Auxin Responds to Flowing Nutrient Solution to Accelerate the Root Growth of Lettuce in Hydroponic Culture

**DOI:** 10.3390/ijms26167742

**Published:** 2025-08-11

**Authors:** Yue Xiang, Jie Peng, Yang Shao, Jung Eek Son, Kotaro Tagawa, Satoshi Yamada, Mina Yamada, Bateer Baiyin, Qichang Yang

**Affiliations:** 1 Research Center for Smart Horticulture Engineering, Institute of Urban Agriculture, Chinese Academy of Agricultural Sciences, Chengdu National Agricultural Science and Technology Center, Chengdu 610213, China; 2 Yazhouwan National Laboratory, Sanya 572025, China; 3 Department of Agriculture, Forestry and Bioresources, Seoul National University, Seoul 08826, Republic of Korea; 4 Faculty of Agriculture, Tottori University, Tottori 680-8553, Japan

**Keywords:** flow condition, hydroponic cultivation, lettuce growth, nutrient solution, root phenotype, urban agriculture

## Abstract

Traditional soil cultivation of lettuce faces challenges; hydroponic technology offers solutions to improve lettuce production. However, the interrelationships among the root phenotype of lettuce, auxin synthesis and signal transduction, and nutrient solution flow, and their effects on hydroponic lettuce growth remain unclear. We investigated the effects of nutrient solution flow state on lettuce’s early growth, transcriptomic changes, and auxin-related gene expression. Growth indicators were measured 2, 4, and 6 days after transplanting. The shoot and root fresh weights, total root length, and root surface area were significantly higher under the flow treatment than under the non-flow condition. The shoot fresh weight increased by 29, 64, and 31%, respectively, at the three growth stages. A clear distinction was observed between the samples from different treatment groups. The Kyoto Encyclopedia of Genes and Genomes (KEGG) pathways that were commonly enriched included “Plant hormone signal transduction (auxin)”. Moreover, the significantly enriched Gene Ontology (GO) terms varied across different time points, which vividly reflected the dynamic characteristics of the plant’s response. Genes related to auxin biosynthesis—such as *AL3F1*, *YUC5*, and *AMI4G*—exhibited higher expression levels under the flow treatment. Overall, these results indicate that nutrient solution flow can promote auxin synthesis and signal transduction in early roots of lettuce.

## 1. Introduction

Lettuce (*Lactuca sativa* L.) is rich in vitamins, minerals, and dietary fiber and is indispensable in human diets [1]. However, with the continuous growth of the global population and rapid urbanization, land resources available for agricultural production have become increasingly scarce. Traditional soil cultivation of lettuce faces a series of problems, such as the gradual decline in soil fertility, frequent pests and diseases, and serious water resource waste [2]. Against this backdrop, hydroponic technology may offer solutions to improve the productivity of lettuce because it is not restricted by soil conditions, enabling the stable production of high-quality products at high yields [3].

The meticulous regulation of environmental conditions to facilitate the optimal growth and development of lettuce under hydroponic cultivation stands as a pivotal issue that captures the attention of both researchers and producers. [4]. The nutrient solution is the main source of nutrients in hydroponics, and its quality and supply directly affect crop growth [5]. The flow characteristics of the nutrient solution influence not only the distribution and transport efficiency of nutrients through the hydroponic system but also the microenvironment around the lettuce roots [6]. The flow of the nutrient solution is a form of mechanical stimulation [7]. In natural environments, plants are often subjected to various forms of mechanical stimulation, such as wind, rain, and animal contact, which interfere with plant growth. However, moderate mechanical stimulation can activate plants’ defense and growth regulation mechanisms, greatly influencing plant morphogenesis, physiological metabolism, and stress resistance [8]. Introducing appropriate mechanical stimulation in the production of hydroponic lettuce is expected to create a beneficial environment for growth.

During plant morphogenesis, auxin, a key regulator of plant growth and development, plays a multidimensional and crucial role [9] and is involved in every aspect of plant growth. In lettuce, auxin plays a key regulatory role in basic physiological processes such as cell elongation, division, and differentiation. It can effectively promote the development of roots, the expansion of leaves, and overall healthy growth of plants [10]. Root morphology is vital for lettuce growth, given its role in the absorption of water and nutrients, and their morphological characteristics, including degree of development, number of branches, length, and surface area, directly affect the efficiency of nutrient absorption from the nutrient solution [11]. Auxin plays a key regulatory role in root growth and development, promoting the elongation and division of root cells and affecting the occurrence and growth of lateral roots [12]. An appropriate concentration of auxin can induce roots to produce more branches and induce root development, thus optimizing root morphology.

The seedling stage of plants is an important period for morphogenesis and establishing physiological functions. This stage is accompanied by the rapid differentiation, formation, and functional initiation of major vegetative organs such as roots, stems, and leaves. In hydroponic lettuce, the perception ability of the root system at the seedling stage in relation to the mechanical stimulus of nutrient solution flow is the core survival strategy for its adaptation to the growth environment, directly determining the optimization of root configuration and the efficiency of resource acquisition [6]. Although numerous studies on the growth regulation of hydroponic lettuce and the physical and chemical properties of nutrient solutions have been conducted, systematic and in-depth research on how mechanical stimulation from the nutrient solution flow causes changes in auxin synthesis and signal transduction in lettuce seedlings and how these changes affect the root growth of hydroponic lettuce is lacking. Additionally, previous studies have failed to comprehensively reveal the interrelationships among the root phenotype of lettuce, auxin synthesis and signal transduction, and nutrient solution flow, as well as their effects on hydroponic lettuce growth.

Therefore, in this study, through hydroponic experiments, we aimed to elucidate the root phenotypes of lettuce grown with and without nutrient solution flow at the seedling stage. By combining transcriptomic data, we explored the molecular mechanisms underlying the process whereby mechanical stimulation of nutrient solution flow affects auxin synthesis and signal transduction in lettuce and changes the root phenotype. The results of this study provide a feasible technical solution for the hydroponic lettuce industry. That is, the findings may help to determine the optimal flow parameters for nutrient solutions used in hydroponic lettuce production. Producers can precisely control the flow rate and mode of the nutrient solution to create an optimal growth environment for lettuce and promote root development and plant growth.

## 2. Results

### 2.1. Effects of Nutrient Solution Flow State on Lettuce Growth

**Figure 1:**
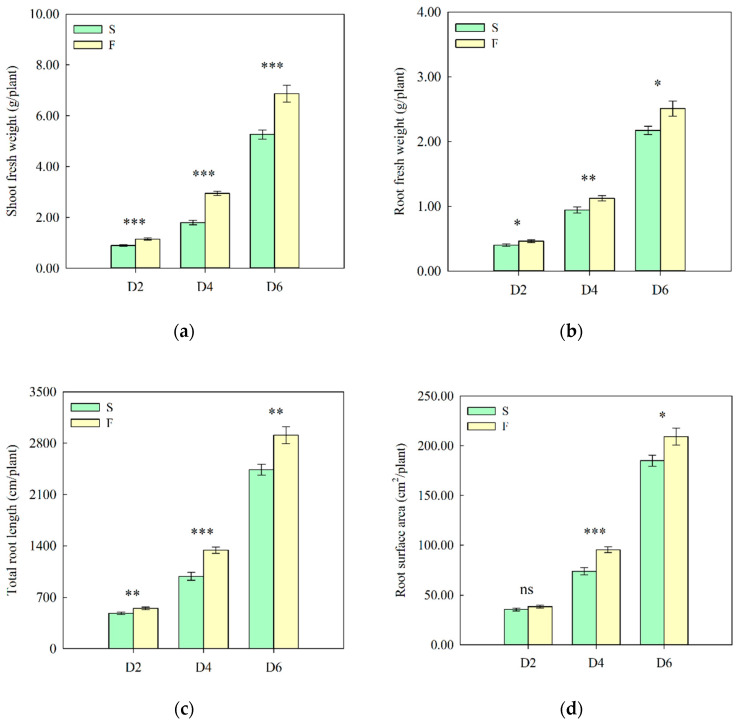

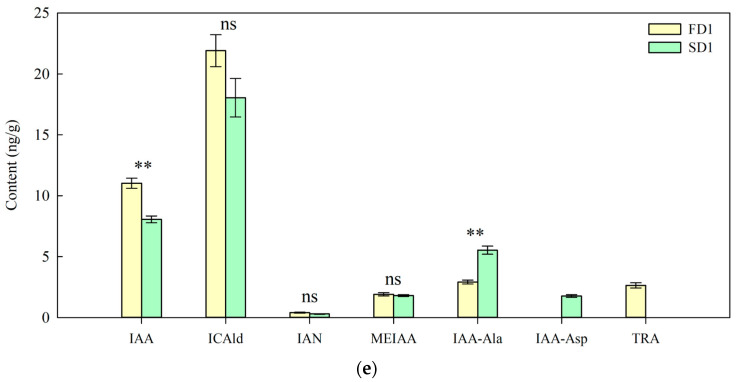
Growth phenotypes and auxin-related metabolites of lettuce under different treatments. (**a**) Shoot fresh weight. (**b**) Root fresh weight. (**c**) Total root length. (**d**) Root surface area. (**e**) Auxin-related metabolite contents of roots. IAA: Indole-3-acetic acid, ICAld: Indole-3-carboxaldehyde, IAN: 3-Indoleacetonitrile, MEIAA: Methyl indole-3-acetate, IAA-Ala: N-(3-Indolylacetyl)-L-alanine, IAA-Asp: Indole-3-acetyl-L-aspartic acid, TRA: Tryptamine; S: 0 L/min, F: 14 L/min; D2, D4, and D6: 2, 4, and 6 days after transplanting, respectively. The data were statistically analyzed using an independent-samples *t*-test, and the results are presented as means ± standard errors (*n* = 4–13). * *p* < 0.05; ** *p* ≤ 0.01; *** *p* ≤ 0.001; ns: no significance.

Lettuce growth under different nutrient solution flow states was measured 2, 4, and 6 days after transplanting. The shoot fresh weight, root fresh weight, total root length, and root surface area of lettuce at D2, D4, and D6 were significantly higher in the flow treatment than those in the non-flow treatment. Compared with the non-flow treatment, the shoot fresh weight in the flow treatment increased by 29, 64, and 31%; the root fresh weight increased by 15, 19, and 16%; the total root length increased by 14%, 36%, and 19%; and the root surface area increased by 8, 29, and 13% at D2, D4, and D6, respectively (Figure 1a–d). Furthermore, we measured the content of auxin in the root systems of hydroponic lettuce under different treatments. The results showed that the content of IAA in the flow treatment was significantly higher than that in the non-flow treatment, and the important precursor substance for IAA biosynthesis, tryptamine, was only detected in the flow treatment (Figure 1e). These results indicate that the flow state of the nutrient solution influenced the growth of roots and shoots during the seedling stage, and the flow treatment was more conducive to root growth and yield improvement.

### 2.2. Transcriptomic Changes in Lettuce Roots Under Different Treatments

**Figure 2:**
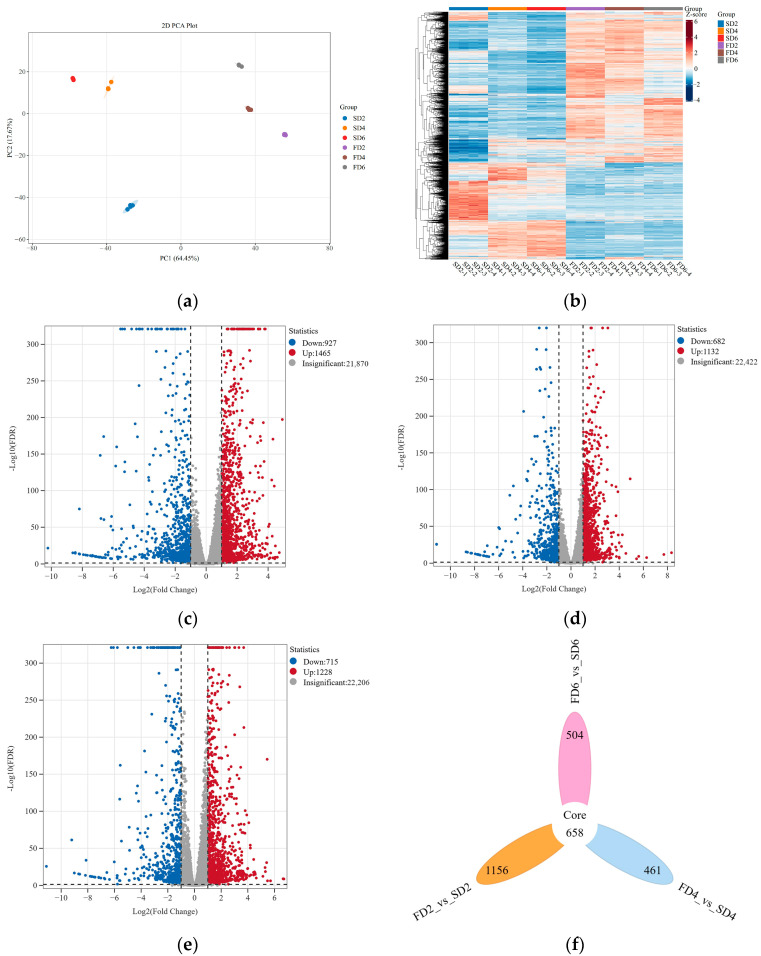

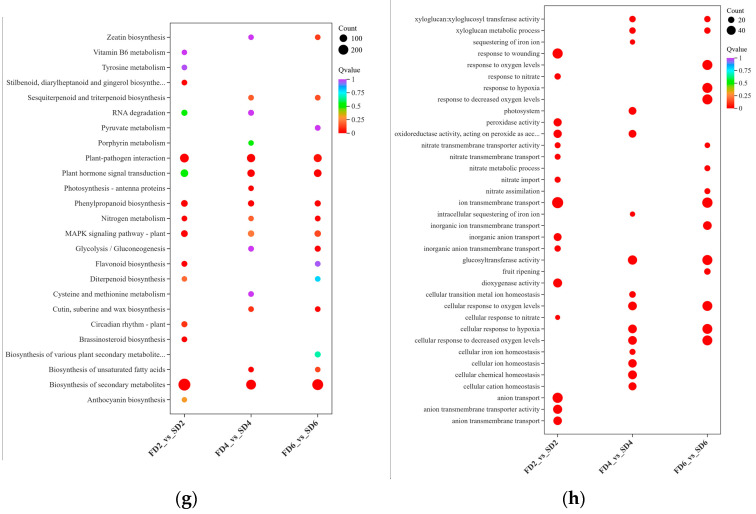
Transcriptomic results under different treatments. (**a**) Principal component analysis. (**b**) Hierarchical clustering analysis. (**c**) Volcano plot of FD2 vs. SD2. (**d**) Volcano plot of FD4 vs. SD4. (**e**) Volcano plot of FD6 vs. SD6. (**f**) Venn diagram of differentially expressed genes (DEGs). (**g**) Kyoto Encyclopedia of Genes and Genomes (KEGG) enrichment bubble chart of DEGs. (**h**) Gene Ontology (GO) enrichment bubble chart of DEGs.

Transcriptomic analysis was conducted on lettuce root samples from six treatments to explore the effects of nutrient solution flow state on gene expression levels in the roots of early-stage lettuce. Clean data (173.2 GB) were obtained from the 24 samples (up to 6 GB per sample). The percentage of Q20 bases was 96% and that of Q30 bases was over 91%; the GC content ranged from 43.11% to 43.67%, with an overall sequencing error rate of 0.03% (Table S1). The results of the principal component analysis showed that the samples from different treatments were distinguishable, and PCA1 and PCA2 could explain 64.45% and 17.67% of the characteristics of the original dataset, respectively (Figure 2a). Hierarchical clustering analysis indicated differences in gene expression patterns under different treatments (Figure 2b).

Differentially expressed genes (DEGs) were screened based on their expression levels. A comparison of FD2 and SD2 revealed 927 downregulated DEGs and 1465 upregulated DEGs (Figure 2c). A comparison of FD4 and SD4 revealed 682 downregulated DEGs and 1132 upregulated DEGs (Figure 2d). A comparison of FD6 and SD6 revealed 715 downregulated and 1228 upregulated DEGs (Figure 2e). Overall, in the three comparison groups, 1156, 461, and 504 unique DEGs were identified, respectively. There were 658 common DEGs among the three comparison groups (Figure 2f).

Gene Ontology (GO) and Kyoto Encyclopedia of Genes and Genomes (KEGG) enrichment analyses were performed on the DEGs of the three comparison groups to investigate the effects of nutrient solution flow state on various biological pathways in lettuce roots. The KEGG enrichment analysis revealed the commonly enriched pathways in the three comparison groups to be “Biosynthesis of secondary metabolites”, “Plant–pathogen interaction,” “Plant hormone signal transduction,” “Phenylpropanoid biosynthesis,” “Nitrogen metabolism,” and “MAPK signaling pathway—plant” (Figure 2g). In FD2 vs. SD2, GO terms such as “Response to wounding,” “Peroxidase activity,” and “Ion transmembrane transport” were significantly enriched (Figure 2h). In FD4 vs. SD4, GO terms such as “Xyloglucan metabolic process,” “Glucosyltransferase activity,” and “Cellular ion homeostasis” were significantly enriched (Figure 2h). In FD6 vs. SD6, GO terms such as “Response to oxygen levels,” “Ion transmembrane transport,” and “Glucosyltransferase activity” were significantly enriched (Figure 2h).

### 2.3. DEGs in the Auxin Biosynthesis and Signal Transduction Pathway

**Figure 3:**
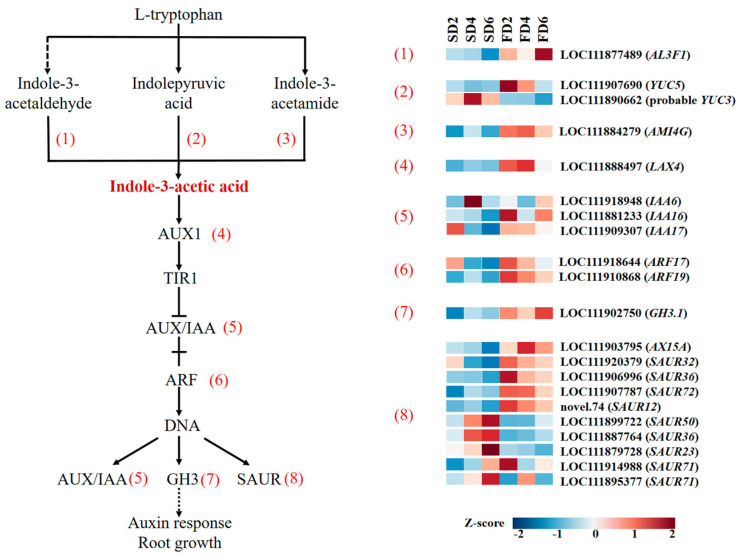
Differentially expressed genes (DEGs) involved in IAA synthesis and signal transduction.

As one of the key plant hormones, the biosynthesis, polar transport, and signal transduction of auxin play crucial roles in regulating the growth and development of plant roots. DEGs in the auxin biosynthesis and signal transduction pathways were analyzed to explore the effects of nutrient solution flow state on the expression levels of auxin-related genes. The expression levels of genes related to auxin biosynthesis, such as *AL3F1*, *YUC5*, and *AMI4G*, were higher in the nutrient solution flow treatment than in the non-flow treatment (Figure 3). In the process of auxin signal transduction, *LAX4*, which encodes auxin transporter-like protein 4, was highly expressed in the FD2 and FD4 treatments; *IAA6*, which encodes auxin-induced protein, was highly expressed in SD4 and FD6; *IAA16* was highly expressed in FD2 and FD6; and *IAA17* had relatively high expression in SD2, FD2, and FD4. The expression of *ARF17* and *ARF19*, which encode auxin response factors, was generally higher in the flow treatment than in the non-flow treatment. *GH3.1*, encoding auxin-responsive GH3-like protein 1, and some genes (*AX15A*, *SAUR32*, *SAUR36*, *SAUR72*, and *SAUR12*) encoding auxin-responsive proteins also showed a similar expression pattern; this means that the expression of these genes was higher in the flow treatment than in the non-flow treatment. These results indicate that the flow of the nutrient solution can promote auxin synthesis and signal transduction in the roots of the seedling-stage lettuce.

### 2.4. Validation by Quantitative Real-Time Reverse Transcription Polymerase Chain Reaction (qRT-PCR)

**Figure 4:**
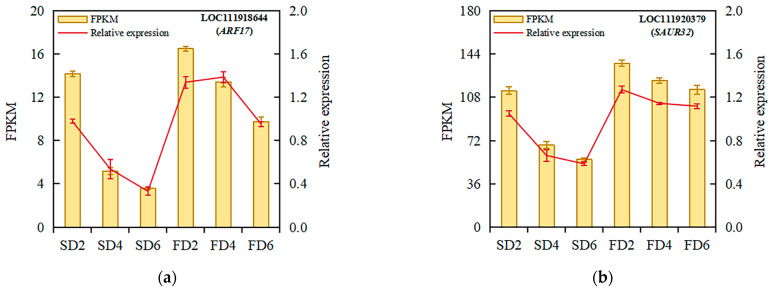

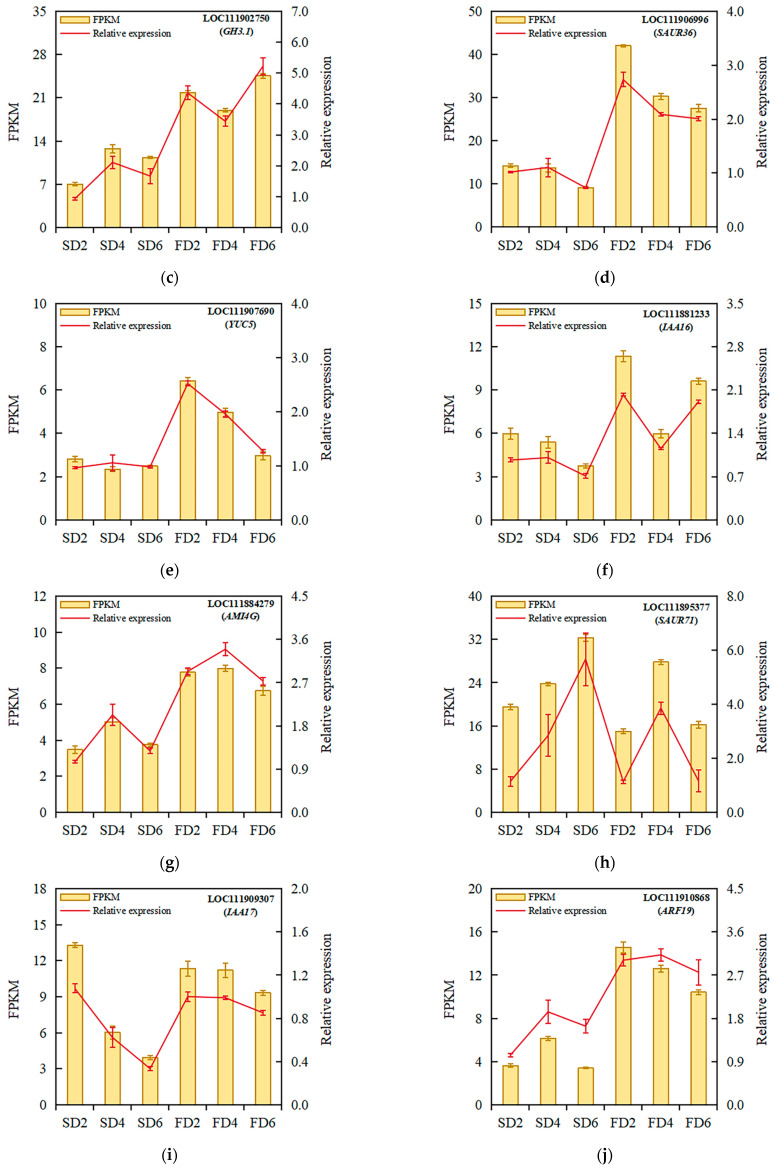
Relative expression analysis of *ARF17* (**a**), *SAUR32* (**b**), *GH3.1* (**c**), *SAUR36* (**d**), *YUC5* (**e**), *IAA16* (**f**), *AMI4G* (**g**), *SAUR71* (**h**), *IAA17* (**i**), and *ARF19* (**j**) under different treatments. Data represent means ± standard errors; fragments per kilobase of transcript per million fragments mapped (FPKM), *n* = 4; relative expression, *n* = 3.

Ten DEGs related to auxin biosynthesis and signal transduction were selected for validation using qRT-PCR to verify the reliability of the transcriptomic analysis results. The validation results showed that the expression trends of the selected genes were consistent with the transcriptomic results (Figure 4), indicating the reliability of the transcriptomic analysis results. The qRT-PCR verification results of the 10 DEGs are presented in Table S2.

## 3. Discussion

In hydroponic lettuce systems, the flow of the nutrient solution can affect lettuce’s growth and nutrient uptake. The shear force generated by the solution flow exerts mechanical stimulation of the roots, thereby altering the morphology. Research has found that compared with non-flow conditions, an appropriate flow rate of the nutrient solution can promote root growth and improve root morphological indicators such as total root length and root surface area, thus facilitating nutrient uptake and increasing aboveground yield [8]. Similar to other environmental signals, the flow of nutrient solution, as a form of mechanical stimulation, can significantly influence the synthesis and signal transduction of auxin in plants and subsequently affect root growth and development.

Auxin, a key plant hormone in plant growth and development, plays an important regulatory role in root growth, morphological development, and plant material accumulation [13]. Auxin regulates root length by modulating the growth of root tip cells. Cells in the root apical meristem can divide continuously. Auxin can activate the expression of relevant genes and promote the synthesis of cell cycle-related proteins, thereby accelerating cell division in the meristematic zone and increasing the number of cells. Simultaneously, auxin can regulate the composition and properties of the cell wall in the elongation zone by increasing the plasticity of the cell wall, enabling cells to elongate under the action of turgor pressure [14]. These effects work together to promote the longitudinal growth of roots, which is beneficial for obtaining more nutrient resources.

Auxin can also regulate signal transduction pathways, enabling pericycle cells to regain their ability to divide, form lateral root primordia, and further develop into lateral roots [15]. Abundant lateral roots lead to an extensive root system in the soil, significantly increasing the contact area with the soil. Auxin is also involved in regulating root hair development [16]. The presence of root hairs can significantly increase root surface area and improve the efficiency of water and nutrient absorption by plant roots.

The results of this study indicated that in the early growth stage of lettuce, the shoot fresh weight, root fresh weight, total root length, and root surface area were higher under the nutrient solution flow treatment than under the non-flow treatment (Figure 1a–d), which is consistent with previous research results. Significant differences were noted in gene expression levels in lettuce roots between the non-flow and flow treatments (Figure 2). Flow treatment promoted the upregulation of more genes, and these DEGs were significantly enriched in the “plant hormone signal transduction” pathway; meanwhile, the content of auxin in the root system of lettuce under the flow treatment was significantly higher than that under the non-flow treatment (Figure 1e). This indicates that the flow of nutrient solution may alter root morphology by regulating the synthesis and signal transduction of auxin in roots, thereby affecting the biomass of both the roots and shoots.

Plant roots perform physiological functions such as absorption, fixation, synthesis, storage, and reproduction. They are indispensable for maintaining normal growth and development [17]. Plant roots can also sense changes in the external environment. The internal genetic system and external environmental stimuli regulate root occurrence and development. Auxin is a hormone that plays a crucial role in developing primary roots, lateral roots, adventitious roots, and root hairs in plants. Mechanical stimulation, a common environmental signal, can significantly influence the synthesis and signal transduction of auxins in plants, affecting root growth and development. Auxin synthesis includes tryptophan-dependent and non-tryptophan-dependent pathways [18]. Tryptophan-dependent pathways are plants’ main source of indole-3-acetic acid (IAA). According to the types of intermediate products, the tryptophan-dependent synthesis pathway is divided into tryptamine, indole-3-pyruvate (IPA), and indole-3-acetamide pathways [18]. Among these, the IPA pathway is one of the most important for plant auxin synthesis.

*YUC* encodes flavin monooxygenase, which catalyzes the conversion of IPA to IAA, and is a rate-limiting gene in the auxin synthesis pathway [19]. Changes in *YUC* expression can affect local auxin biosynthesis, thereby affecting plant growth. After *Arabidopsis thaliana* is subjected to mechanical stimulation, such as touch, the transcription levels of some members of the *YUC* family are significantly upregulated [20]. Flavin monooxygenase is a key enzyme in the tryptophan-dependent auxin synthesis pathway. An increase in its expression will prompt more substrates to be catalyzed to synthesize auxin. This process may involve mechanical stimulation that activates a series of upstream signaling pathways, including calcium and protein kinase signals. These signals are transmitted to the nucleus and bind to cis-acting elements in the promoter region of *YUC*, thereby promoting gene transcription.

When plants are subjected to mechanical stimulation, changes occur in the intracellular environment, such as the membrane potential and ion concentration, affecting auxin signal transduction [21]. Auxin flows between cells in a specific direction, forming a concentration gradient that regulates plant development and environmental response [22]. As a member of the auxin influx carrier family, AUX1 is crucial in multiple plant growth and development processes, including auxin response, gravitropic growth of roots, primary root elongation, and lateral root development [23,24,25].

When auxin synthesis increases, more auxin enters the cells through AUX1 and binds to TIR1/AFB receptors to form a complex. This complex recognizes and binds to AUX/IAA proteins, causing them to be ubiquitinated and degraded via the 26S proteasome, thus relieving the inhibition of ARF transcription factors [26]. ARF is a plant-specific transcription factor with a B3-type DNA-binding domain that directly regulates the expression of early genes by recognizing auxin-responsive elements. Its function depends on the dynamic interaction with AUX/IAA proteins: at low auxin concentrations, ARF forms a heterodimer with AUX/IAA to inhibit the transcription of target genes; when the auxin concentration rises, AUX/IAA is degraded, and ARF activates the expression of target genes [27].

*GH3.1*, a target gene of ARF, regulates IAA homeostasis [28]. Amide synthase, encoded by the early auxin-responsive gene *GH3*, catalyzes auxin binding to amino acids, forming corresponding amino acid complexes. When auxin concentration is extremely high, the GH3 protein catalyzes the binding of auxin to amino acids, and the formed complex acts as an auxin storage pool. When the auxin concentration is extremely low, the auxin–amino acid complex is hydrolyzed by proteases into auxin, which re-enters the auxin signaling pathway, thereby regulating the dynamic balance of auxin in plants.

*SAUR*, another target gene of ARF, can promote plant growth by facilitating cell elongation and regulating plants’ branching angle and curvature [29]. Compared with the non-flow treatment, the flow treatment upregulated genes related to auxin synthesis (such as *YUC5*) and signal transduction (such as *LAX4*, *ARF19*, *GH3.1*, *SAUR36*, and *SAUR72*); this indicates that the mechanical stimulation of nutrient solution flow promotes auxin synthesis and signal transduction, thereby promoting root growth (Figure 3).

In the hydroponic lettuce system, mechanical stimulation from the flowing nutrient solution is non-negligible in promoting auxin synthesis, regulating root morphology, and ultimately promoting lettuce growth. Lettuce roots are continuously subjected to forces of different directions and intensities when the nutrient solution flows. Mechanical stimulation can activate intracellular signaling pathways. When plants are mechanically stimulated, the intracellular calcium ion concentration changes, activating signaling molecules such as calmodulin, which affects the expression of genes related to auxin synthesis [21].

According to our results, mechanical stimulation caused by the flowing nutrient solution can upregulate *YUC* in lettuce roots, promoting auxin synthesis. Changes in auxin levels are crucial for regulating lettuce root morphology. Regarding root elongation, a relatively high level of auxin can promote cell division in the root apical meristem and cell elongation in the elongation zone [30]. Regarding lateral root emergence, auxin is a key signaling molecule for inducing the formation and development of lateral root primordia [15]. The large-scale emergence of lateral roots increased the branching of lettuce roots and expanded the distribution of roots in the nutrient solution. In addition, auxin can promote the growth and development of root hairs [23]. The increase in root hairs significantly expands the surface area of the roots, improving their ability to absorb nutrients from the solution.

The optimization of lettuce root morphology positively influences overall growth. Well-developed and properly shaped roots can more effectively absorb macronutrients such as nitrogen, phosphorus, and potassium and micronutrients such as iron, zinc, and manganese from the nutrient solution, providing sufficient nutrients for lettuce growth. Adequate nutrient supply promotes the growth of the aboveground parts of lettuce, making the leaves lusher and increasing the photosynthetic area, thus improving the photosynthetic efficiency and accumulating more photosynthetic products. The accumulation of photosynthetic products further promotes the growth and development of lettuce, making the stems sturdier and the leaves thicker and significantly improving the quality of lettuce.

Suitable root morphology enhances the adaptability of lettuce to environmental changes. In a hydroponic environment, the roots can better fix the plant, resist the mechanical disturbances caused by factors such as the flowing nutrient solution, and ensure the stability of lettuce growth. Furthermore, well-developed roots improve the ability of lettuce to cope with possible nutrient shortages or other adverse stresses, improve the stress resistance, reduce the occurrence of diseases, and thus increase the yield and quality of lettuce.

During the hydroponic cultivation of lettuce, the appropriate flow of the nutrient solution to increase mechanical stimulation and regulate root morphology by promoting auxin synthesis has multiple benefits for lettuce growth; this is a technical measure that can achieve high-quality and high-yield hydroponic lettuce.

Plant hormones are key signaling molecules regulating plant growth and development. From the perspective of the overall regulatory network of plant growth and development, the process of plant growth is highly complex and sophisticated, involving the regulatory expression of numerous genes and the coordinated action of physiological and biochemical processes [31]. Different hormones interact and influence each other, forming an intricate signaling regulatory network. Apart from auxin, other hormones also play unique roles in this network.

Taking hydroponic lettuce, a modern agricultural production model, as an example, current research has confirmed that appropriately increasing the flow of nutrient solution to enhance mechanical stimulation can promote the synthesis of auxin, thereby regulating root morphology and achieving high-quality and high-yield hydroponic lettuce. However, plant growth is a process affected by multiple factors, and auxin is just one part of it. Other hormones may play an important role in coordinating the growth balance between the aboveground and underground parts, improving photosynthetic efficiency, and optimizing the distribution and utilization of nutrients [30]. For instance, some hormones may be involved in regulating the stomatal opening and closing of lettuce leaves, influencing the absorption of carbon dioxide and transpiration of water, and thus having an impact on photosynthesis and water use efficiency. They may also play a role in the quality formation of lettuce, such as regulating the synthesis of secondary metabolites and affecting the taste, nutritional components, and flavor substances of lettuce [10].

In future research, it is urgent and necessary to systematically conduct research on other hormones. Modern biotechnological means, such as gene-editing technology, transcriptomics, proteomics, and metabolomics, need to be comprehensively applied to comprehensively analyze the synthesis pathways, metabolic networks, and signal transduction mechanisms of other hormones. Through gene-editing technology, genes related to hormone synthesis and signal transduction can be precisely knocked out or over-expressed to study the functions of these genes and the action mechanisms of hormones in plants in depth. Transcriptomics, proteomics, and metabolomics technologies can analyze the changes in gene expression, protein abundance, and metabolites in plants under the action of hormones from a global perspective, revealing the molecular network and physiological and biochemical basis of hormone regulation.

In addition, interdisciplinary research should be carried out, combining theories and methods from multiple disciplines, such as plant physiology, genetics, biochemistry, and fluid mechanics, to deeply explore the regulatory effects of other hormones on plant growth and development under different flow environment conditions [6]. For example, under different conditions of light, temperature, water, and nutrients, research should be conducted on how other hormones respond to nutrient solution flow environment changes and regulate the growth strategies and adaptive mechanisms of plants. At the same time, mathematical modeling method should be used to quantitatively analyze and simulate the hormone regulatory network, predict the effects of hormones under different conditions and the growth dynamics of plants, and provide precise theoretical guidance for agricultural production.

## 4. Materials and Methods

### 4.1. Cultivation Conditions

An indoor hydroponic experiment was conducted, with cultivation conditions for light and temperature implemented according to Baiyin et al. [31]. Lettuce seeds were sown in seedling-raising trays filled with moist vermiculite for 7 days. Healthy seedlings that exhibited consistent growth were transplanted into plastic containers filled with a standard-concentration nutrient solution and cultured under artificial light for 7 days. Lettuce seedlings with relatively consistent growth were selected and planted in the cultivation troughs (1.7 × 0.2 × 0.2 m, length × width × depth). Each trough was filled with 50 L of a standard-concentration nutrient solution with a pH of 6.2 ± 0.1 and an EC of 2.1 ± 0.1. The components and concentrations of the tested nutrient solutions are presented in Table 1. During the cultivation process, LED lights were used for illumination. The light–dark cycle was set to 14:10 h, and the PPFD (Photosynthetic Photon Flux Density) was maintained at 550 umol/m^2^/s. The ambient temperature in the room was maintained at 25 ± 1 °C. In this experiment, two treatments to test nutrient solution flow conditions (S: 0 L/min and F: 14 L/min) and three sampling periods (D2, D4, and D6 for days 2, 4, and 6 after transplantation, respectively) were established, resulting in a total of six treatments (SD2, SD4, SD6, FD2, FD4, and FD6). Each treatment had four replicates—that is, four cultivation troughs—and five plants were planted in each trough.

### 4.2. Determination of Lettuce Phenotypic Indicators

Sampling was performed on days 2, 4, and 6 after transplanting. After sample collection, the shoot and root parts were separated. A root scanner with analysis software (WinRhizo 2013; Regent INS, Quebec, QC, Canada) was used to measure root morphological indicators, such as root length and surface area. After the measurement, an electronic balance (FA2204; Shanghai Lichen Co., Ltd., Shanghai, China) was used to obtain the fresh weights. Root samples for transcriptome analysis were frozen in liquid nitrogen for 15 min after sampling and then stored at −80 °C for later use.

### 4.3. Measurement of Auxin-Related Metabolites in Lettuce Roots

This study employed targeted metabolomics technology based on the UHPLC-MS/MS platform to quantitatively analyze the content of auxins in the root systems of lettuce under different treatments. The pre-treatment steps of the root samples and the chromatography–mass spectrometry acquisition conditions were consistent with the previous study [31]. Each treatment was set up with 4 biological replicates.

### 4.4. Transcriptomic Analysis

Root samples from the six treatments were subjected to transcriptome sequencing, with four biological replicates per treatment. The CTAB method was used to extract RNA from lettuce roots. Subsequently, a Qubit fluorometer and Qsep400 high-throughput biological fragment analyzer were used to measure the concentration and integrity of total RNA. A library was constructed and subjected to quality control following TNA quality inspection. Then, DNA Nanoball was prepared and loaded onto the sequencing chip on the MGI platform. Original data were filtered using fastp [32] to obtain high-quality reads. HISAT2 [33] was used to align the quality-controlled clean reads with the reference genome (https://ftp.ncbi.nlm.nih.gov/genomes/all/GCF/002/870/075/GCF_002870075.4_Lsat_Salinas_v11, accessed on 4 July 2025). The reads for each gene were counted according to the alignment results and positional information of genes in the reference genome. The gene alignment status was calculated using featureCounts [34]. Then, the fragments per kilobase of transcript per million fragments mapped (FPKM) values for each gene were calculated based on the gene length to evaluate the gene expression level. DESeq2 [35] was used for differential expression analysis between the two groups, and the Benjamini–Hochberg method was used to correct the *p*-value to obtain the false discovery rate value. The screening criteria for DEGs were |log2Fold Change| ≥ 1 and false discovery rate < 0.05. The GO and KEGG databases were used for functional annotation and enrichment analysis of the DEGs in the three comparison groups to obtain the KEGG pathways and GO terms significantly enriched with DEGs.

### 4.5. qRT-PCR Analysis

Ten DEGs were selected for qRT-PCR analysis to verify the reliability of the transcriptome data. RNA extracted for transcriptome sequencing was used for qRT-PCR analysis. Complementary DNA synthesis was performed using the Advantage RT-for-PCR Kit (TaKaRa, Tokyo, Japan). The relative expression level of each gene was calculated using the 2^–ΔΔCT^ method [36]. Three biological replicates were used for each treatment. The primer sequences of all tested genes are shown in Supplementary Table S3.

### 4.6. Statistical Analysis

SPSS (version 26; IBM, Chicago, IL, USA) was used to conduct statistical analysis on data such as the shoot fresh weight, root fresh weight, total root length, root surface area, and auxin content. All data were statistically analyzed using an independent-samples *t*-test, and the statistical results are presented as means ± standard errors (*n* = 4–13). Statistical significance was set at *p* < 0.05 (* *p* < 0.05; ** *p* ≤ 0.01; *** *p* ≤ 0.001; ns: not significant).

## Figures and Tables

**Table 1:** Components and concentrations of the nutrient solution.

Composition	Concentration (ppm)
Ca(NO_3_)_2_·4H_2_O	945
KNO_3_	607
NH_4_H_2_PO_4_	115
MgSO_4_·7H_2_O	493
Na_2_Fe(EDTA)	20–40
H_3_BO_3_	2.86
MnSO_4_·4H_2_O	2.13
ZnSO_4_·7H_2_O	0.22
CuSO_4_·5H_2_O	0.08
(NH_4_)_6_Mo_7_O_24_·4H_2_O	0.02

## Data Availability

All data generated or analyzed during this study are included in this published article. The RNA sequencing reads of all samples were submitted to the NCBI Sequence Read Archive (SRA) with the accession code SUB15490731.

## Supplementary Materials

The following supporting information can be downloaded at: https://www.mdpi.com/article/10.3390/ijms26167742/s1.

## Abbreviations

The following abbreviations are used in this manuscript:

DEG	Differentially expressed gene
GO	Gene Ontology
IAA	Indole-3-acetic acid
IPA	Indole-3-pyruvate
KEGG	Kyoto Encyclopedia of Genes and Genomes
