# Supplementary material for: Auxin Responds to Flowing Nutrient Solution to Accelerate the Root Growth of Lettuce in Hydroponic Culture
Source: Int J Mol Sci. 2025 Aug 11;26(16):7742. doi: 10.3390/ijms26167742 (PMC12386315; doi:10.3390/ijms26167742)
Supplement: Supplementary file 1 [file ijms-26-07742-s001.zip › Table S1 All sample summary.pdf]

Table S1 All sample summary

Sample	Group	Raw Reads	Clean Reads	Clean Base (G)	Error Rate (%)	Q20 (%)	Q30 (%)	GC Content (%)
FD2-1	FD2	54800372	54008202	8.10	0.03	96.60	91.59	43.35
FD2-2	FD2	55894442	54952692	8.24	0.03	97.27	93.19	43.33
FD2-3	FD2	46224066	45407690	6.81	0.03	97.06	92.66	43.13
FD2-4	FD2	41500552	40926860	6.14	0.03	96.53	91.46	43.13
FD4-1	FD4	60441670	59295948	8.89	0.03	97.28	93.20	43.39
FD4-2	FD4	52715294	51658622	7.75	0.03	97.15	92.89	43.14
FD4-3	FD4	51977958	51101780	7.67	0.03	97.11	92.78	43.28
FD4-4	FD4	52556500	51420274	7.71	0.03	97.37	93.44	43.20
FD6-1	FD6	50654376	49882238	7.48	0.03	97.11	92.80	43.25
FD6-2	FD6	45552856	44866946	6.73	0.03	96.95	92.39	43.41
FD6-3	FD6	45108658	44380954	6.66	0.03	96.70	91.78	43.25
FD6-4	FD6	51157762	50211338	7.53	0.03	97.12	92.81	43.11
SD2-1	SD2	49203330	48516666	7.28	0.03	97.06	92.66	43.23
SD2-2	SD2	47677592	46961182	7.04	0.03	97.21	93.00	43.67
SD2-3	SD2	51983730	51201278	7.68	0.03	97.01	92.53	43.18
SD2-4	SD2	46113644	45462876	6.82	0.03	97.02	92.60	43.25
SD4-1	SD4	43919934	42827406	6.42	0.03	97.52	93.79	43.11
SD4-2	SD4	45166230	44310508	6.65	0.03	97.16	92.94	43.20
SD4-3	SD4	49640970	48787512	7.32	0.03	97.16	92.90	43.31
SD4-4	SD4	49476468	48637364	7.3	0.03	97.20	92.99	43.22
SD6-1	SD6	42372450	41758516	6.26	0.03	97.15	92.88	43.24
SD6-2	SD6	52485290	51774456	7.77	0.03	96.91	92.17	43.38
SD6-3	SD6	43977584	43362444	6.50	0.03	96.83	92.16	43.27
SD6-4	SD6	43659816	42997040	6.45	0.03	96.74	91.94	43.24
