# Supplementary material for: Auxin Responds to Flowing Nutrient Solution to Accelerate the Root Growth of Lettuce in Hydroponic Culture
Source: Int J Mol Sci. 2025 Aug 11;26(16):7742. doi: 10.3390/ijms26167742 (PMC12386315; doi:10.3390/ijms26167742)
Supplement: Supplementary file 1 [file ijms-26-07742-s001.zip › Table S2 Primer sequences.pdf]

Table S2 Primer sequences

Pri Id	Pri Seq
LOC111881233-F	AAGGTGAGTATGGATGGA
LOC111881233-R	AATAGTGAAGGAACTGAACA
LOC111909307-F	ACGGTGATGAAGAGTAGTA
LOC111909307-R	CTGTTGGTATCCTGTGTAG
LOC111910868-F	TTGAAGATGTTGGTATGAA
LOC111910868-R	GTTGTTGATGTTGTTGAG
LOC111918644-F	ATCTCCTCAAGAAGTTCA
LOC111918644-R	AAGTTAATTAGACGACCATT
LOC111902750-F	TTCCTCACAAGTTCTGGTA
LOC111902750-R	TCTTCGGTCCATCTCTTC
LOC111895377-F	TCTATTCTACAAGGTGTT
LOC111895377-R	GTCCATAATAACCATACC
LOC111906996-F	AACATAGAGTCAGTTCACTT
LOC111906996-R	GATCTAGCCGTCTGTAAC
LOC111920379-F	TCATCATCATCATCATCGTA
LOC111920379-R	CCACCATTACAGCCATAT
LOC111907690-F	TCTATGGCTGGTTGATAA
LOC111907690-R	TCAGTTCTAATGGACCTAA
LOC111884279-F	GCAGATGTGATAGCCTTC
LOC111884279-R	TTCCTTCTCTAACTCTCCAA
actin(nei)-F	ATCCACGAGACGACTTAT
actin(nei)-R	CATCCTATCAGCAATTCCA
