# Supplementary material for: Auxin Responds to Flowing Nutrient Solution to Accelerate the Root Growth of Lettuce in Hydroponic Culture
Source: Int J Mol Sci. 2025 Aug 11;26(16):7742. doi: 10.3390/ijms26167742 (PMC12386315; doi:10.3390/ijms26167742)
Supplement: Supplementary file 1 [file ijms-26-07742-s001.zip › Table S3 Results of qRT-PCR.pdf]

Table S3 Results of qRT-PCR

Genes ID	Treatment	relative expression
LOC111918644	SD2_1	1.00
LOC111918644	SD2_2	0.94
LOC111918644	SD2_3	0.99
LOC111918644	SD4_1	0.71
LOC111918644	SD4_2	0.48
LOC111918644	SD4_3	0.43
LOC111918644	SD6_1	0.29
LOC111918644	SD6_2	0.29
LOC111918644	SD6_3	0.41
LOC111918644	FD2_1	1.42
LOC111918644	FD2_2	1.36
LOC111918644	FD2_3	1.24
LOC111918644	FD4_1	1.32
LOC111918644	FD4_2	1.36
LOC111918644	FD4_3	1.49
LOC111918644	FD6_1	0.94
LOC111918644	FD6_2	1.00
LOC111918644	FD6_3	0.93
LOC111920379	SD2_1	1.00
LOC111920379	SD2_2	1.09
LOC111920379	SD2_3	1.06
LOC111920379	SD4_1	0.77
LOC111920379	SD4_2	0.61
LOC111920379	SD4_3	0.60
LOC111920379	SD6_1	0.56
LOC111920379	SD6_2	0.58
LOC111920379	SD6_3	0.61
LOC111920379	FD2_1	1.24
LOC111920379	FD2_2	1.33
LOC111920379	FD2_3	1.24
LOC111920379	FD4_1	1.14
LOC111920379	FD4_2	1.13
LOC111920379	FD4_3	1.16
LOC111920379	FD6_1	1.09
LOC111920379	FD6_2	1.16
LOC111920379	FD6_3	1.10
LOC111902750	SD2_1	1.00
LOC111902750	SD2_2	0.92
LOC111902750	SD2_3	0.87
LOC111902750	SD4_1	2.46
LOC111902750	SD4_2	2.08

LOC111902750	SD4_3	1.78
LOC111902750	SD6_1	1.35
LOC111902750	SD6_2	1.51
LOC111902750	SD6_3	2.14
LOC111902750	FD2_1	4.67
LOC111902750	FD2_2	4.47
LOC111902750	FD2_3	3.94
LOC111902750	FD4_1	3.78
LOC111902750	FD4_2	3.25
LOC111902750	FD4_3	3.29
LOC111902750	FD6_1	4.73
LOC111902750	FD6_2	5.68
LOC111902750	FD6_3	5.24
LOC111906996	SD2_1	1.00
LOC111906996	SD2_2	1.03
LOC111906996	SD2_3	1.03
LOC111906996	SD4_1	1.44
LOC111906996	SD4_2	0.88
LOC111906996	SD4_3	1.00
LOC111906996	SD6_1	0.74
LOC111906996	SD6_2	0.70
LOC111906996	SD6_3	0.75
LOC111906996	FD2_1	2.63
LOC111906996	FD2_2	2.57
LOC111906996	FD2_3	3.00
LOC111906996	FD4_1	2.16
LOC111906996	FD4_2	2.05
LOC111906996	FD4_3	2.05
LOC111906996	FD6_1	2.08
LOC111906996	FD6_2	1.93
LOC111906996	FD6_3	2.01
LOC111907690	SD2_1	1.00
LOC111907690	SD2_2	0.94
LOC111907690	SD2_3	0.94
LOC111907690	SD4_1	1.33
LOC111907690	SD4_2	1.01
LOC111907690	SD4_3	0.84
LOC111907690	SD6_1	1.01
LOC111907690	SD6_2	0.94
LOC111907690	SD6_3	0.99
LOC111907690	FD2_1	2.59
LOC111907690	FD2_2	2.44
LOC111907690	FD2_3	2.53

LOC111907690	FD4_1	2.07
LOC111907690	FD4_2	1.89
LOC111907690	FD4_3	1.91
LOC111907690	FD6_1	1.26
LOC111907690	FD6_2	1.34
LOC111907690	FD6_3	1.23
LOC111881233	SD2_1	1.00
LOC111881233	SD2_2	0.90
LOC111881233	SD2_3	1.00
LOC111881233	SD4_1	1.19
LOC111881233	SD4_2	0.96
LOC111881233	SD4_3	0.88
LOC111881233	SD6_1	0.67
LOC111881233	SD6_2	0.67
LOC111881233	SD6_3	0.78
LOC111881233	FD2_1	1.99
LOC111881233	FD2_2	2.07
LOC111881233	FD2_3	2.01
LOC111881233	FD4_1	1.18
LOC111881233	FD4_2	1.15
LOC111881233	FD4_3	1.13
LOC111881233	FD6_1	1.95
LOC111881233	FD6_2	1.93
LOC111881233	FD6_3	1.85
LOC111884279	SD2_1	1.00
LOC111884279	SD2_2	1.08
LOC111884279	SD2_3	1.07
LOC111884279	SD4_1	2.47
LOC111884279	SD4_2	1.76
LOC111884279	SD4_3	1.85
LOC111884279	SD6_1	1.19
LOC111884279	SD6_2	1.27
LOC111884279	SD6_3	1.38
LOC111884279	FD2_1	2.96
LOC111884279	FD2_2	3.05
LOC111884279	FD2_3	2.79
LOC111884279	FD4_1	3.66
LOC111884279	FD4_2	3.22
LOC111884279	FD4_3	3.30
LOC111884279	FD6_1	2.85
LOC111884279	FD6_2	2.77
LOC111884279	FD6_3	2.58
LOC111895377	SD2_1	1.00

LOC111895377	SD2_2	0.92
LOC111895377	SD2_3	1.51
LOC111895377	SD4_1	4.39
LOC111895377	SD4_2	2.16
LOC111895377	SD4_3	1.97
LOC111895377	SD6_1	5.74
LOC111895377	SD6_2	3.94
LOC111895377	SD6_3	7.33
LOC111895377	FD2_1	1.23
LOC111895377	FD2_2	1.16
LOC111895377	FD2_3	1.02
LOC111895377	FD4_1	3.68
LOC111895377	FD4_2	3.59
LOC111895377	FD4_3	4.31
LOC111895377	FD6_1	1.86
LOC111895377	FD6_2	1.17
LOC111895377	FD6_3	0.47
LOC111909307	SD2_1	1.00
LOC111909307	SD2_2	1.09
LOC111909307	SD2_3	1.14
LOC111909307	SD4_1	0.80
LOC111909307	SD4_2	0.59
LOC111909307	SD4_3	0.48
LOC111909307	SD6_1	0.33
LOC111909307	SD6_2	0.31
LOC111909307	SD6_3	0.37
LOC111909307	FD2_1	1.09
LOC111909307	FD2_2	0.96
LOC111909307	FD2_3	0.96
LOC111909307	FD4_1	0.97
LOC111909307	FD4_2	1.00
LOC111909307	FD4_3	1.01
LOC111909307	FD6_1	0.83
LOC111909307	FD6_2	0.89
LOC111909307	FD6_3	0.84
LOC111910868	SD2_1	1.00
LOC111910868	SD2_2	1.00
LOC111910868	SD2_3	1.10
LOC111910868	SD4_1	2.42
LOC111910868	SD4_2	1.75
LOC111910868	SD4_3	1.64
LOC111910868	SD6_1	1.43
LOC111910868	SD6_2	1.55

LOC111910868	SD6_3	1.93
LOC111910868	FD2_1	2.78
LOC111910868	FD2_2	3.10
LOC111910868	FD2_3	3.15
LOC111910868	FD4_1	3.31
LOC111910868	FD4_2	3.17
LOC111910868	FD4_3	2.89
LOC111910868	FD6_1	2.24
LOC111910868	FD6_2	3.12
LOC111910868	FD6_3	2.91
